# Effects of Plasma Treated Alumina Nanoparticles on Breakdown Strength, Partial Discharge Resistance, and Thermophysical Properties of Mineral Oil-Based Nanofluids

**DOI:** 10.3390/ma14133610

**Published:** 2021-06-28

**Authors:** Norhafezaidi Mat Saman, Izzah Hazirah Zakaria, Mohd Hafizi Ahmad, Zulkurnain Abdul-Malek

**Affiliations:** Institute of High Voltage & High Current, School of Electrical Engineering, Universiti Teknologi Malaysia, Johor Bahru 81310, Johor, Malaysia; hafezaidimatsaman@gmail.com (N.M.S.); izzahhazirah13@gmail.com (I.H.Z.); zulkurnain@utm.my (Z.A.-M.)

**Keywords:** mineral oil, alumina nanofluids, atmospheric pressure plasma, partial discharge

## Abstract

Mineral oil has been chosen as an insulating liquid in power transformers due to its superior characteristics, such as being an effective insulation medium and a great cooling agent. Meanwhile, the performance of mineral oil as an insulation liquid can be further enhanced by dispersing nanoparticles into the mineral oil, and this composition is called nanofluids. However, the incorporation of nanoparticles into the mineral oil conventionally causes the nanoparticles to agglomerate and settle as sediment in the base fluid, thereby limiting the improvement of the insulation properties. In addition, limited studies have been reported for the transformer oil as a base fluid using Aluminum Oxide (Al_2_O_3_) as nanoparticles. Hence, this paper reported an experimental study to investigate the significant role of cold plasma treatment in modifying and treating the surface of nano-alumina to obtain a better interaction between the nano-alumina and the base fluid, consequently improving the insulation characteristics such as breakdown voltage, partial discharge characteristics, thermal conductivity, and viscosity of the nanofluids. The plasma treatment process was conducted on the surface of nano-alumina under atmospheric pressure plasma by using the dielectric barrier discharge concept. The breakdown strength and partial discharge characteristics of the nanofluids were measured according to IEC 60156 and IEC 60270 standards, respectively. In contrast, the viscosity and thermal conductivity of the nanofluids were determined using Brookfield DV-II + Pro Automated viscometer and Decagon KD2-Pro conductivity meter, respectively. The results indicate that the 0.1 wt% of plasma-treated alumina nanofluids has shown the most comprehensive improvements in electrical properties, dispersion stability, and thermal properties. Therefore, the plasma treatment has improved the nanoparticles dispersion and stability in nanofluids by providing stronger interactions between the mineral oil and the nanoparticles.

## 1. Introduction

Since 1892, mineral oil has been used as an insulation medium due to its excellent insulating properties and has effectively served as a dielectric coolant [1]. One of the reasons that mineral oils have been chosen as a transformer oil is the ability to transfer heat more effectively than solid-based insulating materials, where solid insulations typically have issues, such as containing void impurities and poor thermal conductivity. Besides, mineral oil also has improved self-healing after failure, making it suitable for power transformer insulation [2]. Previously, significant progress had been made in improving the electrical breakdown strength and heat transfer of mineral oils by dispersing a certain number of nanoparticles into the mineral oil to create a liquid called nanofluid or nano-transformer oil [3]. The main purpose of exploring nanofluids is to enhance the insulation and thermal properties, consequently prolonging the power transformer’s lifespan and minimizing the pre-breakdown phenomenon.

According to the literature, multiple studies have been conducted to determine nanofluids’ thermal conductivity and viscosity. Ahmadi et al. [4] conducted a comprehensive study to compare various machine learning approaches in modeling the dynamic viscosity of nanofluid. It was found that an increase in the concentration of nanoparticle causes higher dynamic viscosity [4]. In contrast, an increment in the temperature reduces the value of dynamic viscosity [4]. Besides, the new approach of enhanced artificial neural network (EANN) was developed by Bagherzadeh et al. [5] to predict the thermal conductivity of hybrid nanofluids. In addition, Peng et al. [6] highlighted that the artificial neural network might estimate the nanofluid thermal conductivity with high accuracy. This method is useful to identify the effectiveness of various input parameters that might affect the nanofluids’ thermal properties. Ghasemi et al. [7] also pointed out that the neural network was good predictors in identifying the thermal conductivity of nanofluid. The study revealed that increasing the solid volume fraction of nanoparticles would increase the thermal conductivity properties of nanofluids [7].

Giwa et al. [8] recommended using hybrid ferrofluids for engineering applications because they possess lower viscosity than mono-particle ferrofluids and nanofluids. Moreover, hybrid nanofluids are also capable of improving electrical conductivity and reduction in viscosity [8]. Alrashed et al. [9] also conducted a comprehensive study to determine carbon-based nanofluids’ thermophysical properties. The results showed that using nanoparticles in a reasonable volume of fractions has improved the working fluids’ thermal conductivity and viscosity [9]. Furthermore, Mansour et al. [10] reported that a small amount of alumina nanoparticles increased the heat transfer of the alumina-mineral oil-based nanofluids.

The electrical insulation properties such as partial discharge and breakdown strength were also enhanced by incorporating the nanoparticles into the base fluids. This has been proven by numerous studies conducted, such as Jin et al. [11] that investigated the properties of mineral oil-based silica nanofluids, and the results showed that the addition of SiO_2_ nanoparticles had improved the alternating current (AC) breakdown voltage of the mineral oil. Furthermore, Sridhara et al. [12] reported that alumina nanofluid could also increase a transformer oil’s dielectric strength and life. Besides, Fan et al. [13] prepared a transformer oil nanofluid by dispersing TiO_2_ nanoparticles, and they found an improvement of 15% to 43%, higher than that of pure transformer oil for the mean value of breakdown voltage.

Jin et al. [14] studied the partial discharge (PD) behavior of mineral oil and silica oil-based nanofluids, and the outcome shows that silica/mineral oil nanofluids obtained 20% higher inception voltage and the total discharge magnitude had been reduced, compared to using pure mineral oil. In addition, Prasad et al. [15] performed a study on the effect of silica nanoparticles on partial discharge characteristics of FR3 transformer oil. The results presented in the work showed that the partial discharge inception voltage and the stable PD formation voltage had been significantly improved for nano-silica/FR3 oils compared to the natural ester oil (FR3). On top of that, Makmud et al. [16] also investigated the characteristics of partial discharge of transformer oil-based Fe_2_O_3_ nanofluids under AC voltage. They found that the PD inception voltage (PDIV) of their nanofluids had increased, compared to pure transformer oil.

Unfortunately, the addition of nanofillers or nanoparticles in base fluids may result in sedimentation in nanofluids due to their surface incompatibility. Moreover, the incompatibility between the surface of the nanoparticles and base fluid causes the nanoparticles to agglomerate, thereby limiting the improvement of the insulation properties. Thus, the trends of studies have focused primarily on the technique to improve the dispersion and compatibility between the nanoparticles and the base fluids. Conventionally, previous researchers have introduced surfactants or dispersants to address sedimentation problems in the nanofluids, but the functionality of the surfactants at high temperatures is also a major concern, especially for high-temperature applications. Typically, introducing surfactants could involve chemical solvents that are difficult to be decomposed, have high toxicity, and require a complex handling process to incorporate the nanoparticle and the base fluid. Even though the surfactants approach can avoid premature sedimentation, the precise number of surfactants required to maximize nanofluids’ insulation properties remains a question. The most suitable and effective type of surfactants is also the main challenge in overcoming the agglomeration issue [17,18].

Recently, plasma discharge has been introduced as a surface modification technique to improve the compatibility in nanomaterials application. Plasma is a state of matter formed through the photoionization process that can be exhilarated using high voltage sources. Previously, plasma treatment has been utilized to improve the compatibility between the nanoparticles and solid-based polymer. Likewise, the main purpose of this treatment is to overcome the agglomeration of the nanoparticle and obtain a uniform dispersion of the nanoparticles into the polymer matrix. Plasma treatment is typically used to functionalize a certain chemical functional group on the surface of nanoparticles, which effectively anchors a new covalent bond during the mixing process of nanoparticles into base fluids. Besides, the plasma treatment is also useful to strengthen the covalent bond of the nanoparticles by producing radical species such as an oxygen-containing compound and a hydroxyl group. Thus, the combination of plasma treatment and nanoparticles seems to have a great outcome in terms of the insulation properties’ enhancement, such as an increase in the breakdown strength and partial discharge resistance of the nanofluids due to the advantages and benefits of this technique. Plasma is also an alternative method of the treatment process that does not require a chemical solvent to functionalize the surface of the nanoparticles. Therefore, this could be another reason for choosing plasma as a modification technique.

Previously, Musa et al. [19] pointed out that atmospheric pressure plasma treatment was effective in forming uniform dispersion of nanoparticles within the polymer matrix. Besides, this technique was also attributed to forming strong covalent bonds with the molecules of the base insulating materials [19]. Yan et al. [20] also conducted a comprehensive study to investigate the dielectric breakdown strength of epoxy resin filled with atmospheric pressure plasma-treated nano-silica. The results showed that plasma treatment was a great technique to improve the compatibility of the nanoparticles and the polymer matrix, improving the dielectric breakdown strength. Furthermore, Awang et al. [21] revealed that the partial discharge resistance of nanomaterials was highly achievable by treating the nanofillers with cold plasma compared with the pure insulating materials.

The implementation of plasma treatment on the nanoparticles in preparing nanofluid seems necessary to explore intensively since this technique has great potential to improve the dispersion of nanoparticles, indirectly enhancing the insulation and thermal properties of the nanofluids. Currently, a limited number of studies have been conducted regarding the performance of plasma treatment in improving the properties of nanofluids. Thus, it seems necessary to fill this gap by exploring the effectiveness of plasma treatment in modifying the surface morphology of nano-alumina before it is dispersed into mineral oil.

In this work, cold atmospheric pressure plasma treatment was used to treat Al_2_O_3_ nanoparticles to enhance the AC breakdown strength, viscosity properties, and thermal conductivity of nanofluids using helium gas as discharge working gas. Some researchers tackled agglomeration problems in nano-mixtures by using thermal non-equilibrium atmospheric-pressure plasma to change the nano-silica surface [22]. Meanwhile, other researchers claimed that plasma-treated nanoparticles with the desired surface functionality could interact strongly with liquid molecules that are better dispersed into the base fluid to form a stable suspension [23]. The interaction and compatibility between alumina nanoparticles and mineral oil can be improved using the cold atmospheric pressure plasma method.

Hence, this paper introduces plasma-treated nano alumina into mineral oil-based nanofluids to enhance the AC breakdown strength and partial discharge resistance and boost two important thermophysical properties: viscosity and thermal conductivity. Nano transformer oil has the potential for better heat transfer characteristics relative to conventional transformer oils used for cooling purposes [24].

## 2. Materials and Methods

The base fluid used in this work was Hyrax Hypertrans mineral oil supplied by Hyrax (Klang, Malaysia), which is good as a dielectric and coolant. It has a density of 0.895 g/mL and a minimum dielectric strength of 30 kV. The Alumina (Al_2_O_3_) nanofiller was purchased from Sigma Aldrich (Petaling Jaya, Malaysia) with an average particle size of 13 nm. Nanofluid samples were prepared with 0.01 wt%, 0.05 wt%, 0.1 wt%, and 0.3 wt%, respectively. In this work, plasma was applied to the nanoparticles for surface modification to prevent early sedimentation in oil and improve the dielectric properties. The alumina nanofillers were treated using atmospheric pressure plasma in the plasma chamber that applied the dielectric barrier discharge (DBD) concept. These plasma discharges were applied on the alumina nanofiller to form functionalized surfaces of major reactive species.

**Figure 1:**
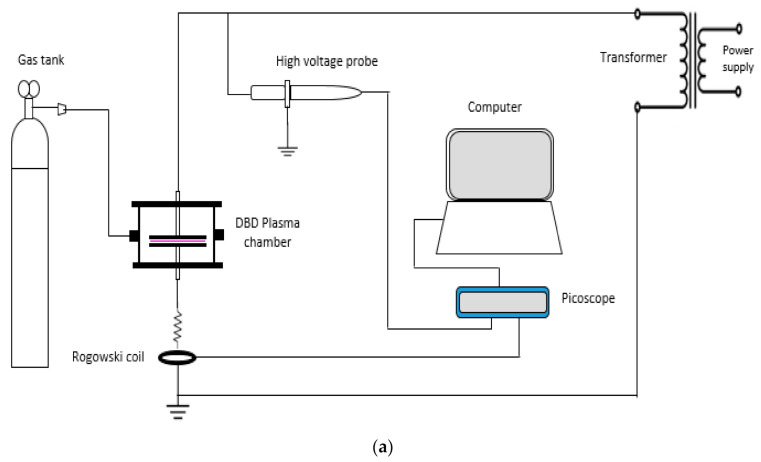

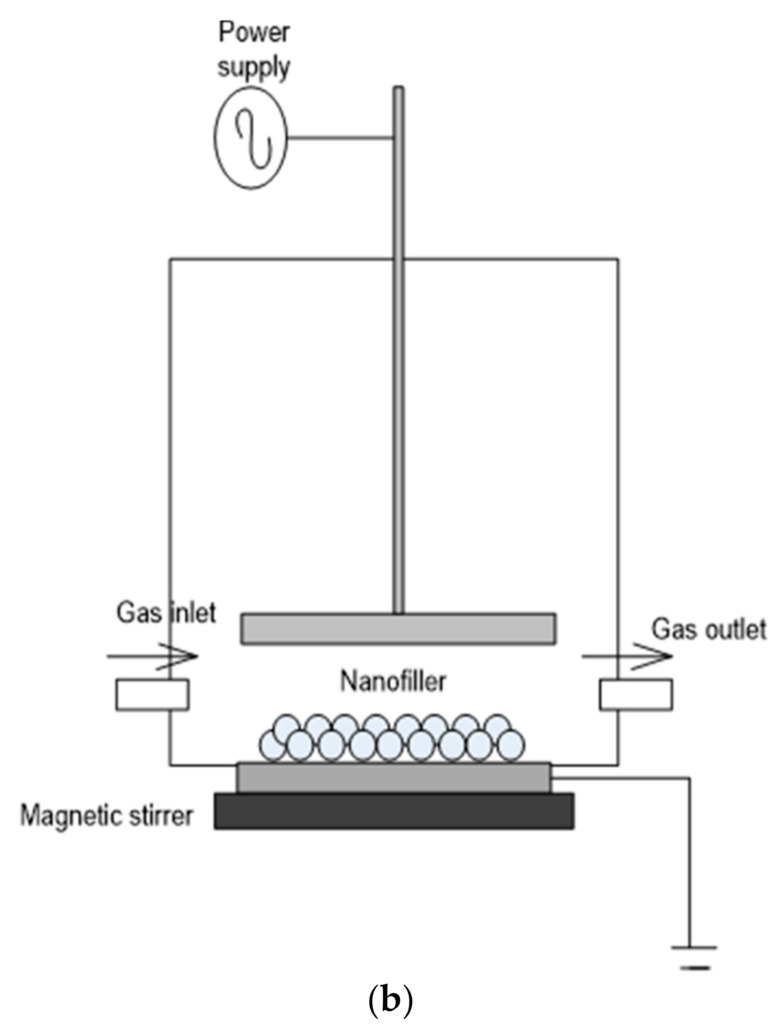
Plasma experimental setup: (**a**) Schematic diagram of plasma treatment setup, (**b**) setup of the DBD plasma chamber.

The plasma discharges were applied on the surface of the nanoparticles to avoid early sedimentation in the oil. A 50 Hz power supply produced the cold atmospheric air pressure plasma with a maximum of 10 kVrms of applied voltage, and the output power consumed was 9 to 10 W. The plasma setup consisted of two glass plates, and the nanoparticles were placed on the plates. A tin-coated copper coil electrode was mounted 2 mm above the top of the nanoparticles layer. Helium gas was used as the working gas for discharge. The duration of the treatment was 30 min. The nanoparticles were stirred for 30 s for every 5 min of surface treatment to obtain a homogenous plasma treatment as recommended and indicated in the reference [12]. The schematic diagram of the experimental setup for dielectric barrier discharge (DBD) plasma treatment is shown in Figure 1a, while the setup for the DBD chamber is shown in Figure 1b. After the treatment, the treated nanoparticles were combined with the base oil according to the two-step method.

**Figure 2:**
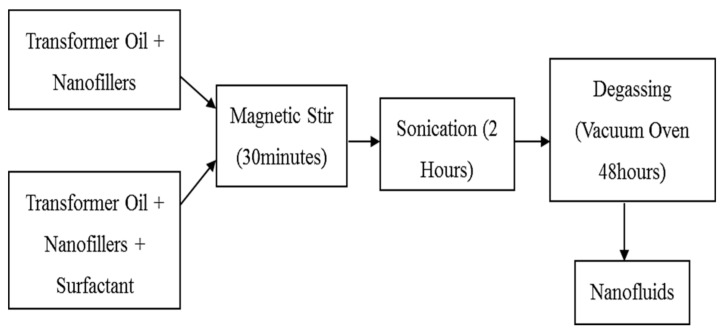
Flowchart for the preparation of mineral oil-based alumina nanofluids.

The two-step method is the most economical method of producing nanofluids on a broad scale, as nanoparticles’ synthesis techniques have already been scaled up to industrial production standards [24]. In this experiment, as shown in Figure 2, the two-step method was used to prepare mineral oil-based nanofluid, where the nanoparticles were first weighed. Nanoparticles were initially distributed in mineral oil. The mixture was stirred within 30 min with a magnetic stirrer and then sonicated to ensure good dispersion of the mixtures. Next, the alumina nanofillers with different concentrations of 0.3 wt%, 0.1 wt%, 0.05 wt%, and 0.01 wt% mass fraction were added to the 100% by weight of mineral oil to study the impact of nanoparticles concentration. The samples were then dried in a vacuum oven at 60 ℃ for at least 24 h [11].

## 3. Results

### 3.1. AC Breakdown Voltage

**Figure 3:**
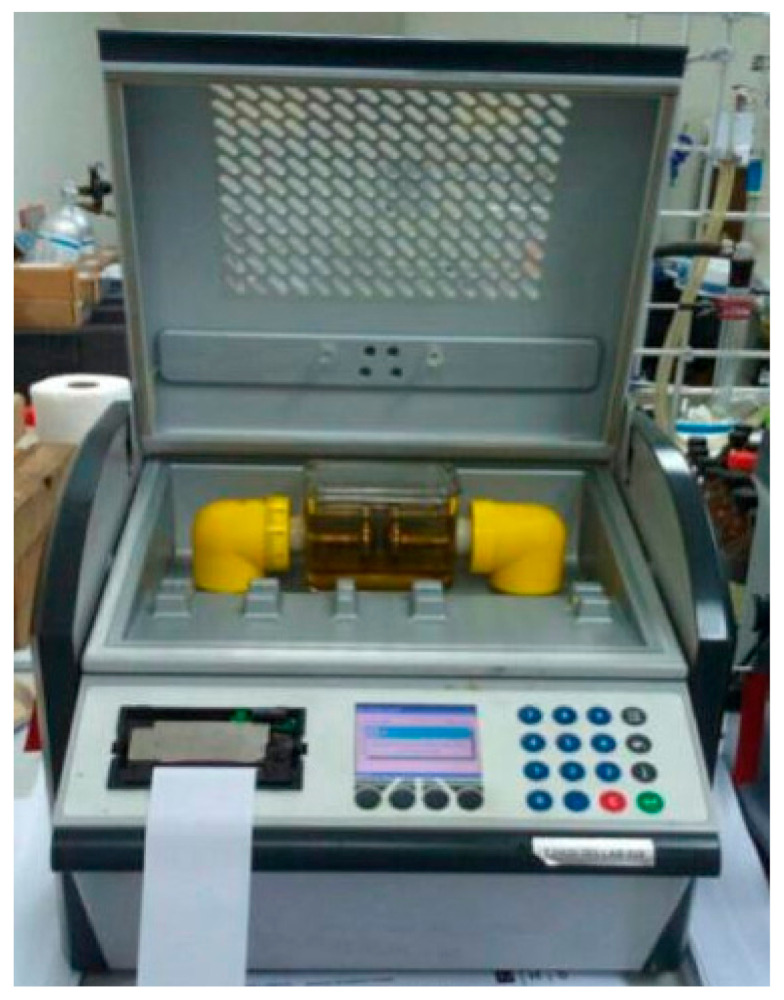
Oil test set.

The breakdown voltage test was carried out in compliance with the IEC 60156 standard. The configuration of the electrode consists of two spherical brass electrodes with a gap of 2.5 mm. The AC voltage (50 Hz) with an increased rate of 20 kV/s was applied until a breakdown occurs. Three sets of six measurements of breakdown tests were recorded for each type of nanofluid. The results were analyzed using Weibull analysis. Figure 3 shows the oil test set to run the AC breakdown voltage for alumina nanofluids.

**Figure 4:**
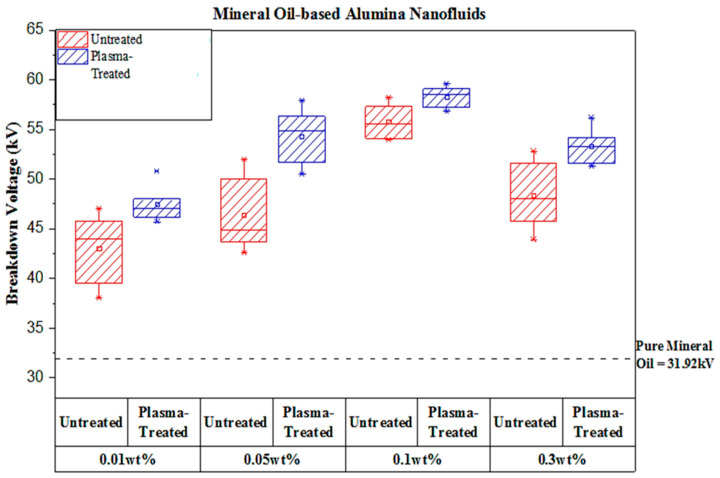
Average AC breakdown voltages of mineral oil-based plasma-treated and untreated alumina nanofluids.

**Figure 5:**
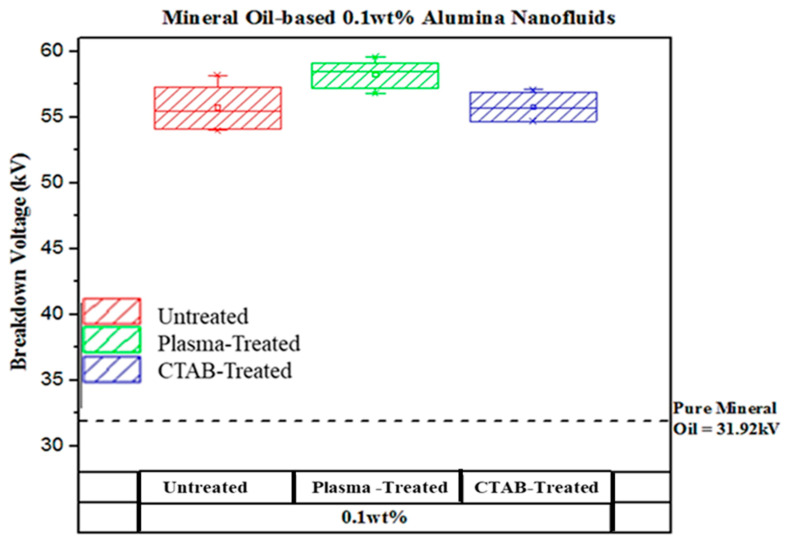
Average AC breakdown voltages of mineral oil-based with 0.1 wt% of plasma-treated, untreated, and CTAB-treated alumina nanofluids.

Figure 4 shows the average AC breakdown voltage for mineral oil-based plasma-treated and untreated alumina nanofluids. Meanwhile, the average AC breakdown voltage for mineral oil-based 0.1 wt% of plasma-treated, untreated, and cetyl trimethylammonium bromide (CTAB) treated alumina nanofluids is shown in Figure 5. The results showed 48.37 kV, 55.77 kV, 46.35 kV, and 43.05 kV for untreated alumina nanofluid of 0.3 wt%, 0.1 wt%, 0.05 wt%, and 0.01 wt% samples, respectively. Previously, Kong et al. [25] and Tendero et al. [26] carried out work to modify alumina nanoparticles using the atmospheric pressure plasma treatment method.

The plasma-treated alumina nanofluid showed 53.30 kV, 58.28 kV, 54.35 kV, and 47.47 kV for 0.3 wt%, 0.1 wt%, 0.05 wt%, and 0.01 wt% samples, respectively. For comparing purposes, the plasma-treated alumina nanofluid obtained the highest result compared to the CTAB-treated and untreated nanofluid samples for 0.1 wt%, as illustrated in Figure 5. The overall enhancement of the AC breakdown voltage for alumina nanofluids relative to pure mineral oil can be seen in Table 1. Plasma-treated nanofluids have a higher increased AC breakdown voltage than pure mineral oil, untreated nanofluids, and CTAB-treated nanofluids. However, the results have shown that the AC breakdown voltage for all nanofluids is higher than the pure mineral oil, which is in line with the outcomes obtained by Yuzhen et al. [27], Zhou et al. [28], and Du et al. [29].

### 3.2. Weibull Analysis

**Figure 6:**
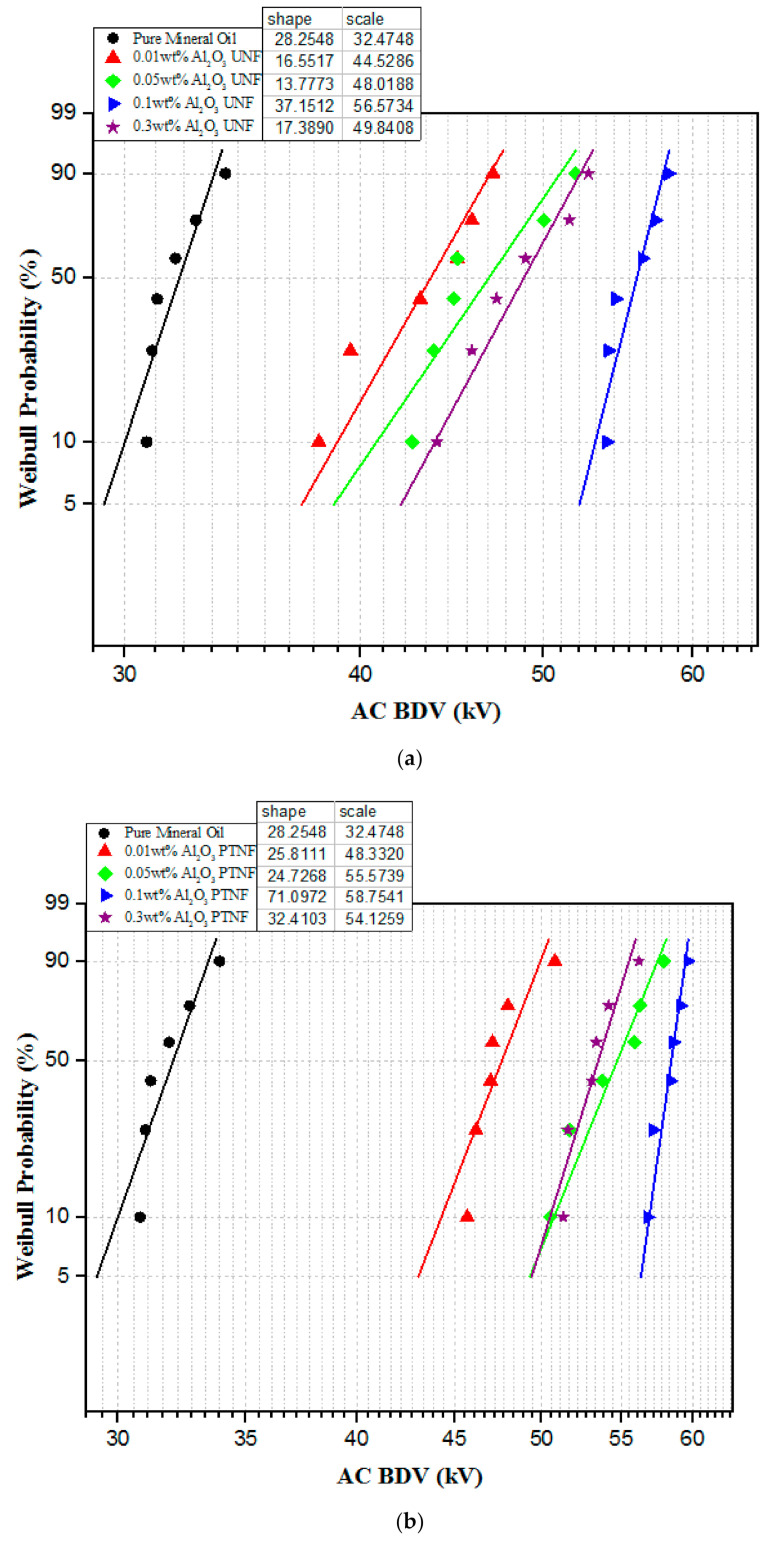

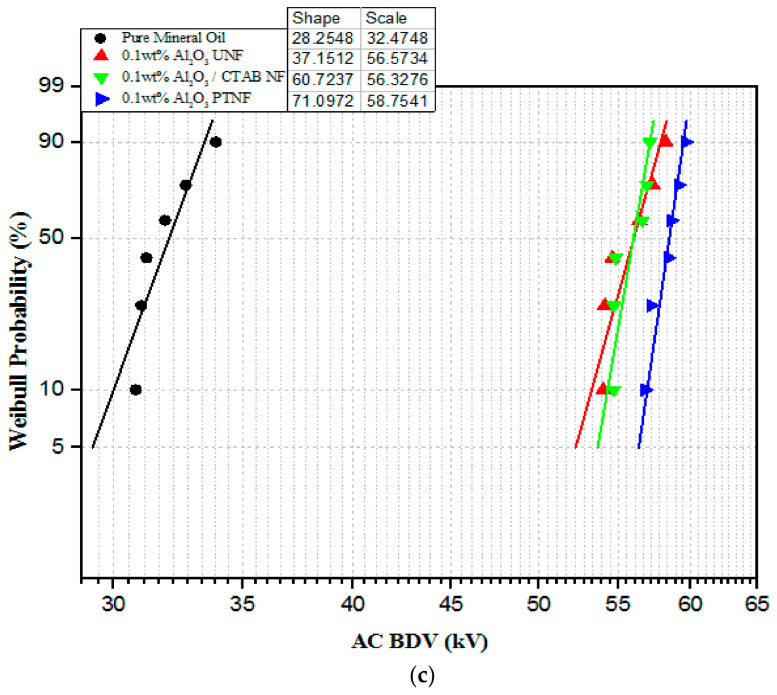
Weibull analyses of AC breakdown voltages; (**a**) MO + Al_2_O_3_ untreated nanofluids, (**b**) MO + Al_2_O_3_ plasma-treated nanofluids, (**c**) all samples of 0.1 wt% Al_2_O_3_ nanofluids.

Weibull analysis can be used to estimate the breakdown voltage obtained from AC breakdown data for lower failure probabilities with two-parameter functions [30]. For example, Figure 6a shows the two-parameter Weibull analysis of the AC breakdown voltage of pure mineral oil and mineral oil-based untreated alumina nanofluids with 95% confidence intervals. At the same time, the Weibull analysis method of AC breakdown voltage for mineral oil-based plasma-treated alumina nanofluids is shown in Figure 6b.

**Figure 7:**
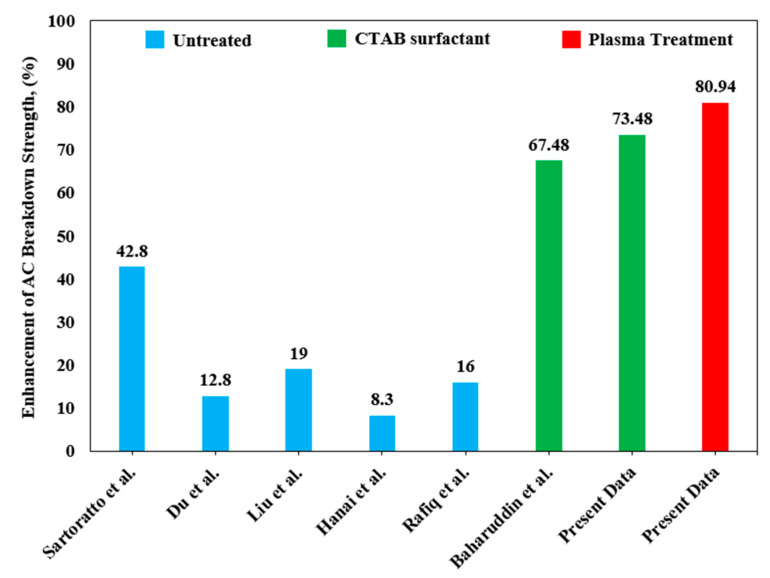
Comparison of the present experimental data of AC breakdown voltage with previous findings.

Moreover, alumina nanoparticles could significantly increase the AC breakdown voltage of the mineral oil. The enhancement of the breakdown voltage at a concentration of 0.1 wt% nanoparticle is remarkable. The AC breakdown voltage of Al_2_O_3_ nanofluids increases with the increase in particle concentration. Since 0.1 wt% of both untreated and treated alumina nanofluid samples show the highest breakdown voltage in each category, one type of 0.1 wt% alumina nanofluid containing the surfactant (CTAB) was used for comparison purposes. The nanofluid with the addition of surfactant was used in this experiment to determine if it contributes a great deal to the breakdown voltage. As illustrated in Figure 6c, it is clear that 0.1 wt% alumina nanofluid with CTAB indicates the lowest voltage value of 56.33 kV, and the plasma-treated alumina sample has the highest breakdown voltage value. Therefore, the results obtained from the Weibull analysis had about the same value as their mean voltage. The summary of scale and shape parameters of breakdown voltage results for alumina nanofluids from the Weibull probability analysis are shown in Table 2. Figure 7 shows the comparison between the present experimental data and the previous findings of the AC breakdown strength.

Table 3 depicts the detail of previous studies according to the type of nanofluid and the effective loading of nanoparticle in enhancing the AC breakdown strength. It is shown that the plasma treatment implemented in the present work is the most promising technique that effectively improves the AC breakdown voltage of the nanofluids incorporated with 0.1 wt% of nano-alumina, followed by the CTAB surfactant technique, which also increased the AC breakdown voltage by more than 50% of the pure mineral oil. Moreover, the variation of nanoparticles used with different effective loadings of nanoparticles exhibits less than a 50% improvement.

### 3.3. Partial Discharge Characteristics

**Figure 8:**
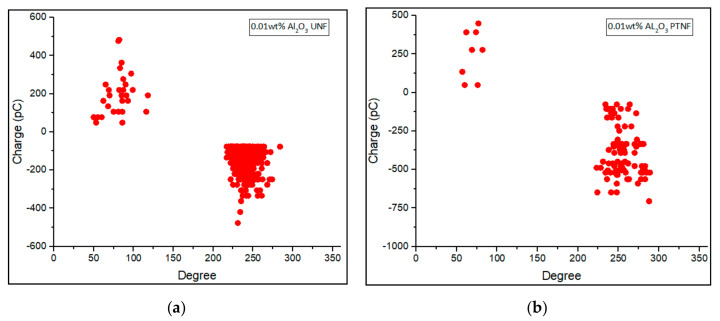
Phase—resolved PD patterns of (**a**) 0.01 wt% Al_2_O_3_ UNF and (**b**) 0.01 wt% Al_2_O_3_ PTNF.

**Figure 9:**
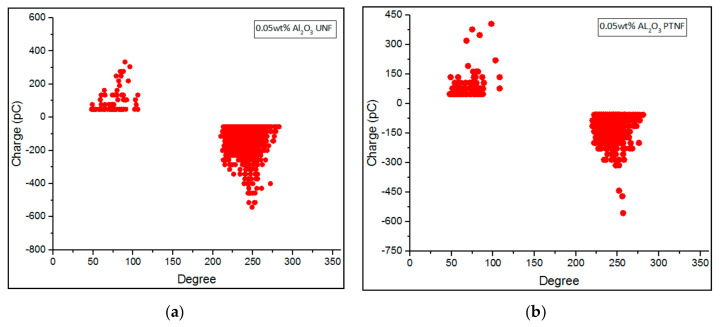
Phase—resolved PD patterns of (**a**) 0.05 wt% Al_2_O_3_ UNF and (**b**) 0.05 wt% Al_2_O_3_ PTNF.

**Figure 10:**
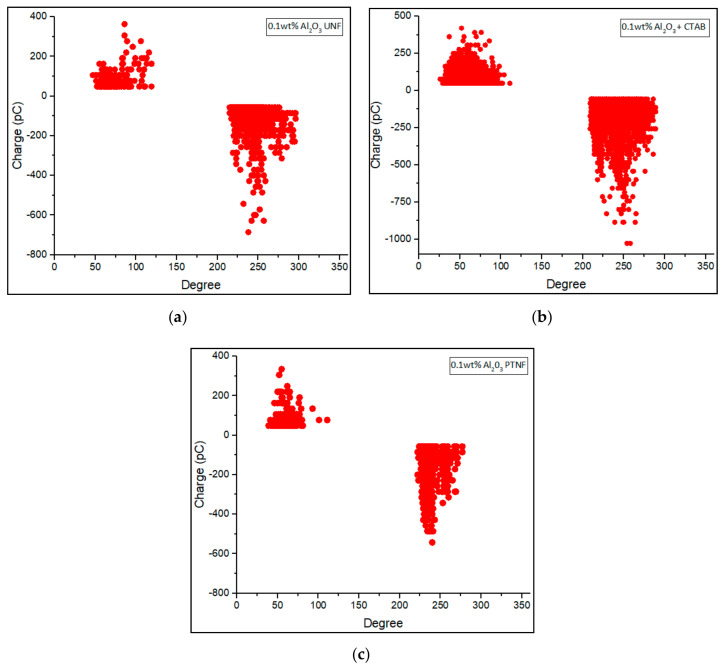
Phase—resolved PD patterns of (**a**) 0.1 wt% Al_2_O_3_ UNF, (**b**) 0.1 wt% Al_2_O_3_ + CTAB NF, and (**c**) 0.1 wt% Al_2_O_3_ PTNF.

**Figure 11:**
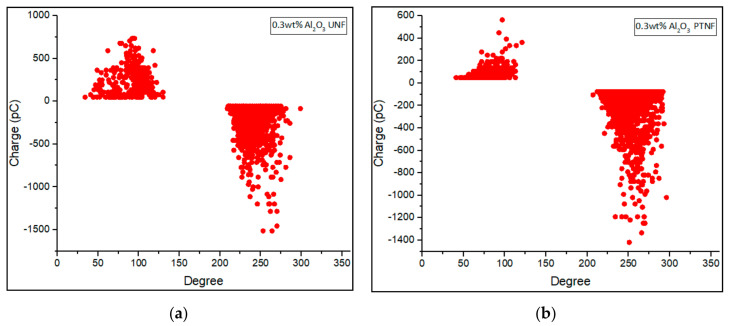
Phase—resolved PD patterns of (**a**) 0.3 wt% Al_2_O_3_ UNF and (**b**) 0.3 wt% Al_2_O_3_ PTNF.

**Figure 12:**
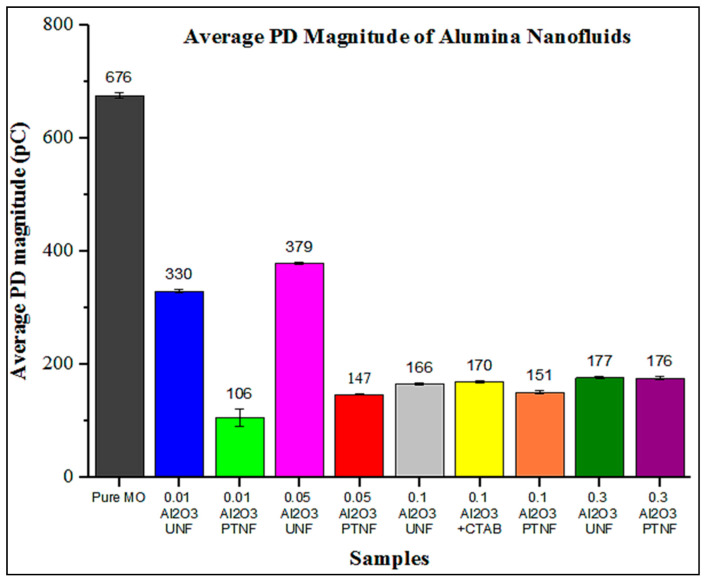
Average PD magnitude (pC) of all alumina nanofluid samples.

Phase-resolved PD patterns of 0.01 wt%, 0.05 wt%, 0.1 wt%, and 0.3 wt% of Al_2_O_3_ nanofluids were shown in Figure 8, Figure 9, Figure 10 and Figure 11, respectively. Discharge occurrences in mineral oil were repeatable in both positive and negative half-cycles. In addition, only one or two discharge pulses were produced during the power cycle. Furthermore, the polarity effect can also be shown by the discharge in the mineral oil, which tends to have much more negative pulses than the positive pulse in the cycle, as also claimed in the previous research by Makmud et al. [16]. Meanwhile, Figure 12 shows the average PD magnitude for all alumina nanofluid samples. As illustrated in the figure, pure mineral oil shows the highest average PD magnitude, at about 676 pC, while the lowest is shown by 0.01 wt% plasma-treated alumina nanofluid, which is about 106 pC. The other samples obtained a lower average PD magnitude compared to pure mineral oil, and this trend is in line with the previous studies by Makmud et al. [16] and Nagendran et al. [36]. Likewise, plasma-treated nanofluid samples show a lower average PD magnitude compared to the untreated samples. For 0.01 wt% alumina nanofluids, the plasma-treated sample has a lower average PD magnitude of about 106 pC than the untreated sample at about 330 pC.

Furthermore, for 0.05 wt% nanofluids, the average PD magnitude charge values are about 379 pC and 147 pC, respectively, for untreated and plasma-treated samples. As usual, a sample with surfactant (CTAB) was used for comparison purpose. The 0.1 wt% has been determined to be mixed with surfactant owing to the fact that this amount resulted in a higher AC breakdown strength. With 0.1 wt% alumina and surfactant, the sample exhibited a higher value of average PD magnitude, at about 170 pC, compared to untreated and plasma-treated samples at about 166 pC and 151 pC, respectively. Lastly, for 0.3 wt% samples, the untreated and plasma-treated nanofluids show an average PD magnitude of about 177 pC and 176 pC, respectively. These results demonstrate that the addition of alumina nanoparticles into the mineral oil would certainly enhance its PD characteristics, as also revealed by Muangpratoom et al. [37], Mohamad et al. [38], and Jacob et al. [39].

**Figure 13:**
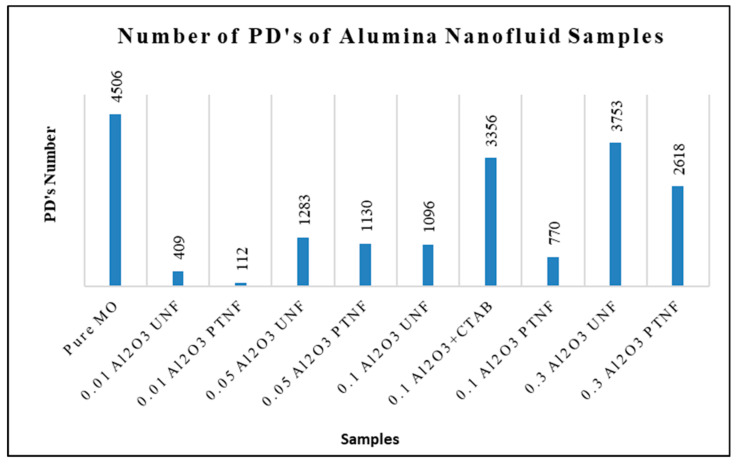
The number of PDs of all alumina nanofluid samples.

**Figure 14:**
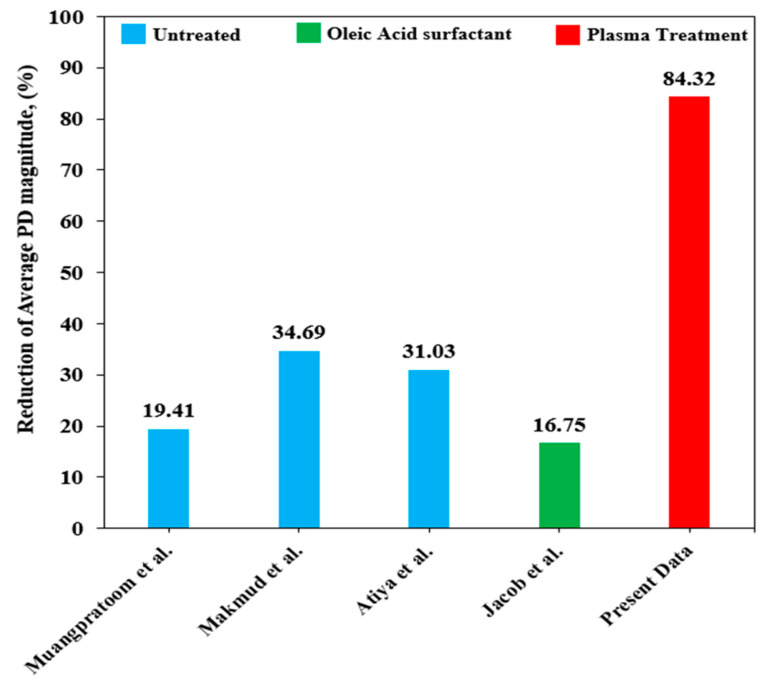
Comparison of the present experimental data of average PD magnitude with previous findings.

The total number of partial discharges (PDs) of all alumina nanofluid samples is illustrated in Figure 13. The highest PDs number is shown by pure mineral oil, which is about 4506, while the lowest value is shown by 0.01 wt% plasma-treated alumina nanofluid, which is about 112. The lower the number of PDs, the better the sample, but the average of the PD magnitude must be emphasized. Figure 14 shows the comparison between present experimental data and previous findings of the average PD magnitude.

Table 4 represents the detail of previous findings regarding the type of nanofluids and effective loading of nanoparticles to minimize the PD magnitude. Significantly, the plasma treatment on the surface of nanoparticles effectively improves the PD endurance, which proved that incorporating 0.01 wt% of plasma-treated alumina into mineral oil reduced the average PD magnitude of pure mineral oil by 84.32%. However, compared to other results obtained in previous findings, all the configurations of untreated and surfactant nanoparticles only showed less than a 34.69% reduction of the PD magnitude.

### 3.4. Viscosity

**Figure 15:**
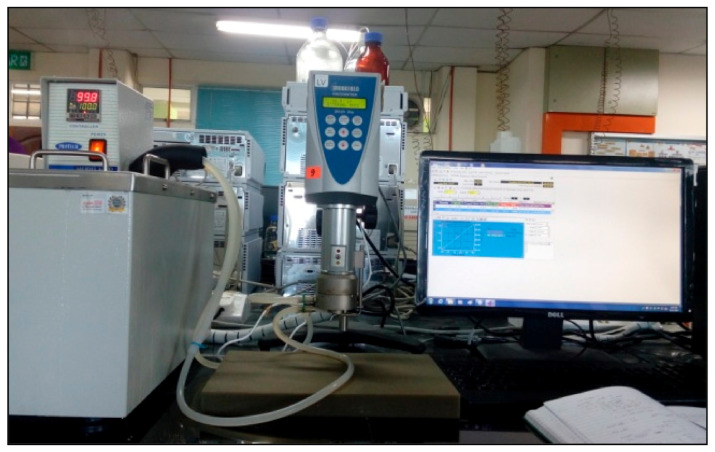
Brookfield DV-II + Pro Automated viscometer with CP-41 spindle.

Viscosity is one of the most critical parameters studied since it can influence both the nanofluids’ heat transfer and electrical properties [24]. Viscosity testing was performed on the oil samples using the Brookfield DV-II + Pro Automated viscometer, manufactured by Brookfield Engineering Laboratories (Middleboro, MA, USA) as shown in Figure 15, using the CP-42 spindle based on the ISO 3104 standard [41]. In this paper, the rheometer measured the viscosity at temperatures of 40 °C and 60 °C.

**Figure 16:**
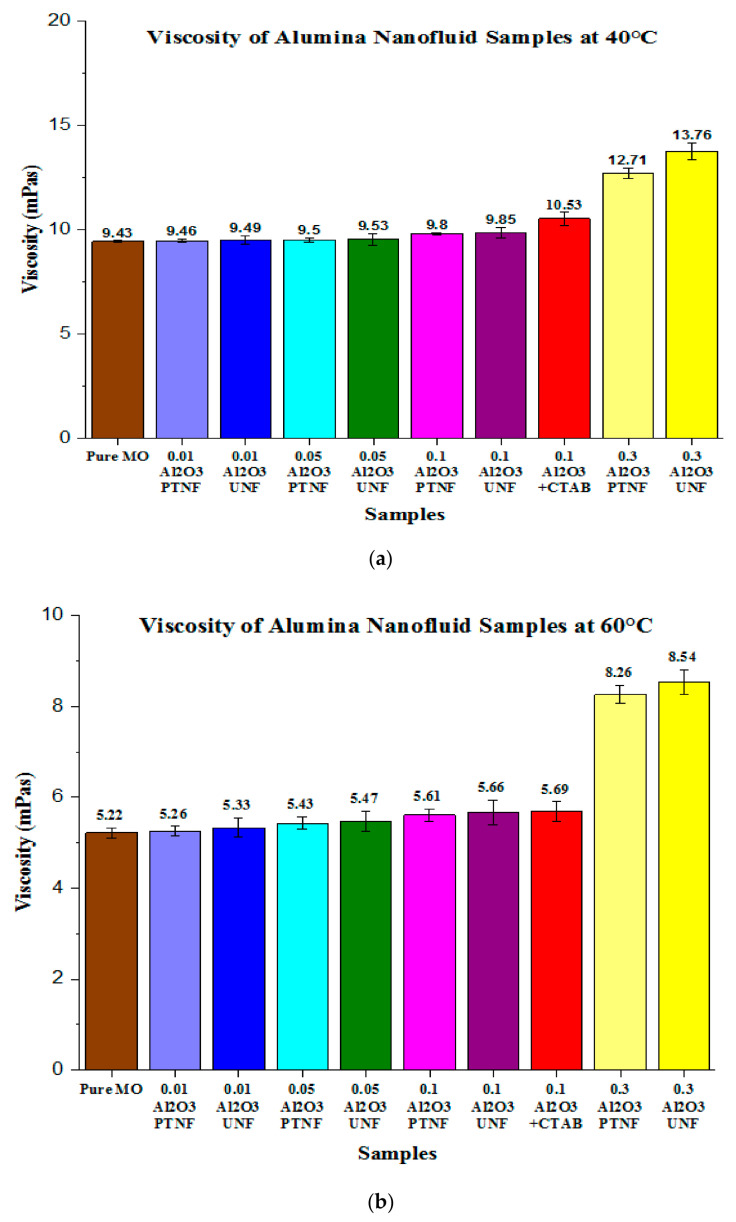
Average viscosity of pure MO and Al_2_O_3_ nanofluid samples at (**a**) 40 °C and (**b**) 60 °C.

Figure 16a shows the average viscosity of Al_2_O_3_ nanofluid samples at 40 °C. The alumina nanofluids showed that the higher the concentration of nanoparticles, the higher the viscosity of the sample. This was also collectively revealed by Yu et al. [42] and Wong et al. [43]. It was clearly shown that the sample with the highest percentage of nanoparticles, which was 0.3 wt%, resulted in the highest viscosity readings for both untreated and plasma-treated samples with values of about 13.76 mPas and 12.71 mPas, respectively.

The alumina nanofluid with CTAB was used for comparison purposes. With the value of 10.53 mPas, the sample with CTAB did not aid much in reducing the viscosity of nanofluids. The value showed a significant change compared to 0.1 wt% alumina UNF with a value of about 9.85 mPas, while the 0.1 wt% alumina PTNF showed a value of 9.80 mPas. Between these three 0.1 wt% samples, the plasma-treated sample was still the best, with a value of 9.80 mPas; 0.01 wt% alumina UNF and 0.01 wt% alumina PTNF are 9.49 mPas and 9.46 mPas, respectively. In contrast, 0.05 wt% alumina UNF and 0.05 wt% alumina PTNF values are 9.53 mPas and 9.50 mPas, respectively.

The viscosity results of alumina nanofluids at 60 °C, as shown in Figure 16b, show that the higher the concentration of nanoparticles, the higher the sample’s viscosity. The 0.3 wt% is the highest percentage of nanoparticles used, showing the highest viscosity readings for both untreated and plasma-treated samples with the values of 8.54 mPas and 8.26 mPas, respectively. The same as at 40 °C, the sample with surfactant (CTAB) was used for comparing 0.1% nanoparticles with untreated and plasma-treated samples. With a value of 5.69 mPas, the sample with surfactant did not help much in reducing the viscosity of nanofluids. The value indicates no substantial difference relative to the untreated 0.1 wt% with a value of 5.66 mPas, while the plasma-treated 0.1 wt% indicates a value of 5.61 mPas.

The 0.01 wt% alumina UNF and 0.01 wt% alumina PTNF samples, respectively, gave the values of 5.33 mPas and 5.26 mPas. Meanwhile, 0.05 wt% alumina UNF and 0.05 wt% alumina PTNF samples obtained values of 5.47 mPas and 5.47 mPas, respectively. As mentioned before, lower-concentration nanoparticles have a negligible impact on the viscosity of mineral oil. As illustrated in Figure 16, both untreated and plasma-treated alumina nanofluids at 0.01 wt% and 0.05 wt% did not show any significant changes due to a smaller concentration of nanoparticles having a negligible effect on the viscosity of mineral oil [4].

### 3.5. Thermal Conductivity

Dispersing nanoparticles in fluids is an efficient way to improve the thermal conductivity of nanofluids. In this paper, the relationship between thermal conductivity and viscosity of mineral oil-based alumina nanofluids in a temperature range from 40 °C to 80 °C under different nanoparticles weight fractions of 0.01 wt%, 0.05 wt%, 0.1 wt%, and 0.3 wt% were addressed.

**Figure 17:**
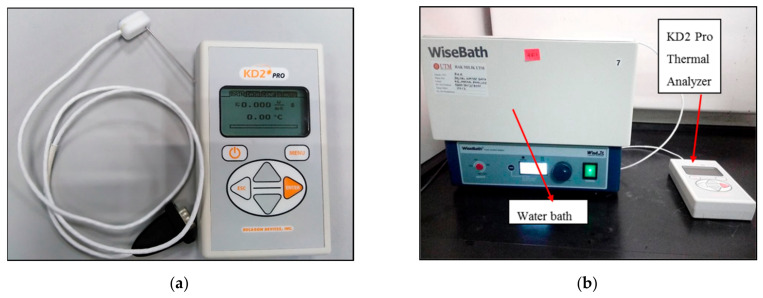
Thermal conductivity instruments: (**a**) Thermal Properties Analyzer, Decagon KD2-Pro Conductivity meter with KS-1 sensor, (**b**) Wise water bath used to conduct thermal conductivity test.

**Figure 18:**
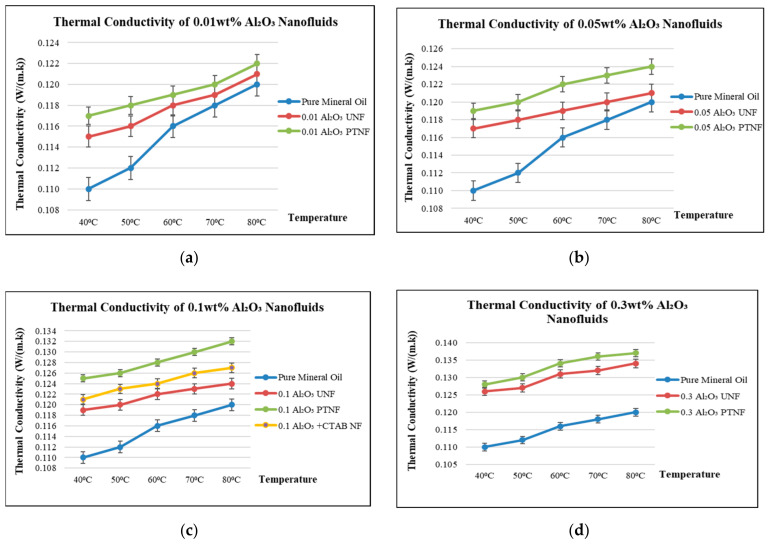
Thermal conductivity results: (**a**) 0.01 wt% Al_2_O_3_ nanofluids, (**b**) 0.05 wt% Al_2_O_3_ nanofluids, (**c**) 0.1 wt% Al_2_O_3_ nanofluids, and (**d**) 0.3 wt% Al_2_O_3_ nanofluids.

Thermal conductivity and viscosity are the essential thermophysical properties of any nanofluids affecting their heat transfer performance. Temperature and volume fractions significantly affect the thermal conductivity and viscosity characteristics of mineral oil-based alumina nanofluids. In this paper, the researchers used a KD2-Pro thermal property analyzer with a heating element and thermistor on the KS-1 sensor needle for thermal conductivity measurements, as shown in Figure 17a. Hence, to keep the temperature within the range, a water bath, as shown in Figure 17b, helped to heat and maintain the temperature value required for the thermal purpose. Figure 18a–d shows that the thermal conductivity of alumina nanofluids increased with an increase in nanoparticle concentrations at elevated temperatures compared to pure mineral oil, and these results agreed with the conclusion made by Xiang et al. [44]. The 0.1 wt% alumina with CTAB nanofluid has higher thermal conductivity than pure mineral oil and untreated nanofluids but shows lower thermal conductivity than the plasma-treated nanofluid.

### 3.6. TEM Analysis

**Figure 19:**
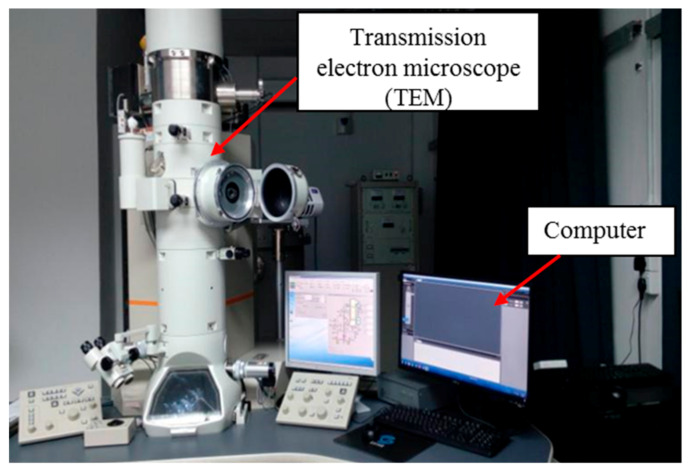
JEOL JEM-2100F field emission electron microscope.

The structure of the sample and dispersion of the nanofillers within the sample can be observed and further studied using JEOL JEM-2100F transmission electron microscope (TEM), manufactured by JEOL Ltd. (Akishima, Tokyo, Japan) as such in Figure 19. Furthermore, the sample preparation of nanofluids for TEM analysis is easy and not complicated as only a small drop of nanofluids was placed onto a TEM grid and was allowed to dehydrate at room temperature. For sample preparation for the transmission electron microscope, nanofluid samples are first mixed with distilled water, with a ratio of 1:10 in volume [45].

**Figure 20:**
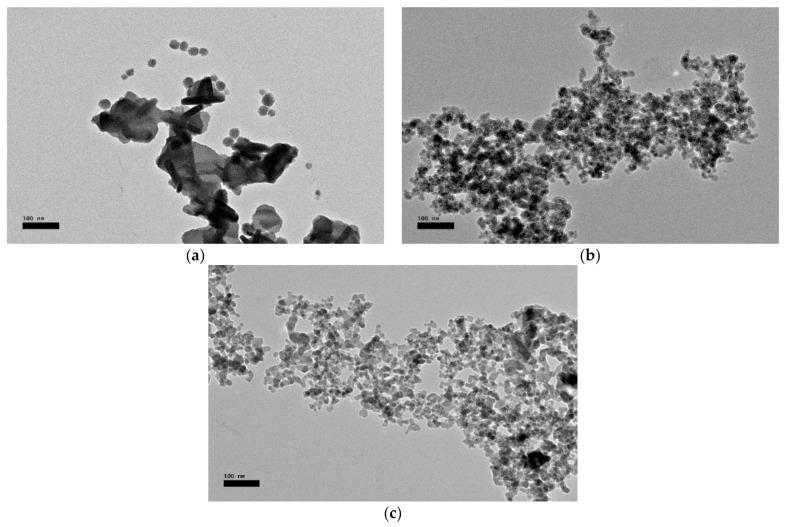
TEM images: (**a**) 0.1 wt% Al_2_O_3_ with CTAB nanofluids, (**b**) 0.1 wt% untreated Al_2_O_3_ nanofluids, and (**c**) 0.1 wt% plasma-treated Al_2_O_3_ nanofluids. The inserted scale bar is 100 nm.

Using the CTAB as a surfactant, the alumina nanofluids with surfactant were observed to have a high level of agglomeration, as shown in Figure 20a. Meanwhile, in Figure 20b, without any chemical treatment in an untreated nanofluid, the size of agglomerated particles and the number of primary particles in a nanoparticle cluster was significantly decreased. However, the stirrer and sonication do not seem to be an effective method to break down the size of the alumina nanoparticle clusters. As shown in Figure 20c, the plasma treatment was the most effective method to deagglomerate the alumina nanoparticle dispersions in mineral oil.

## 4. Discussion

In this work, there are two types of mineral oil-based nanofluids prepared, namely plasma-treated mineral oil-based nanofluids and untreated mineral oil-based nanofluids. However, a CTAB-treated nanofluid was also prepared with 0.1 wt% nanoparticles for comparison purposes. These four types, including pure mineral oil, were compared to their performances for AC breakdown voltage, partial discharge, viscosity, and thermal conductivity characteristics. The weight percentage of alumina added into the mineral oil were 0.01, 0.05, 0.1, and 0.3 wt% only. Meanwhile, the weight percentage of CTAB added was 0.075 wt% of the alumina nanofluids [35].

For AC breakdown tests, both 0.1 wt% plasma-treated alumina nanofluids indicate the highest breakdown voltage than other samples. The Weibull analyses also show the same trend as AC breakdown voltage results. Ionization in cold atmospheric pressure plasmas is not very high but nevertheless very effective in generating high concentrations of reactive radicals, as many researchers have chosen this approach to modify the surfaces of nanoparticles. Consequently, the plasma surface modification treatment of alumina nanoparticles has led to higher AC breakdown voltage. The 0.1 wt% plasma-treated nanofluids have the highest enhancement in about 45.25% compared to pure mineral oil. Such apparent breakdown voltage enhancement based on the addition of plasma-treated nanoparticles has suggested a significant solution to improve the breakdown voltage of the mineral oil, which has been reported to decrease after specific years of service [46]. It is noteworthy that the results obtained indicate that this simple plasma treatment method can enhance the interfacial interaction, thus increasing the breakdown voltage of the nanofluids. The enhancement behavior of AC breakdown voltage with increased nanoparticles concentration can be explained due to the relaxation time constant and polarization of nanoparticles in the nanofluids. This was also explained by Wang et al. in reference [47]. In addition, the polarization produces charges that can change the potential distribution around the nanoparticles in the nanofluids. After that, dielectric nanoparticles’ polarization changes them into potential wells necessary to capture free electrons [48]. The breakdown strength of transformer nanofluids is often correlated with additional traps from the dispersed nanoparticles, which work to capture electrons and reduce the energy of electrons travelling through the transformer oil. This mechanism was believed and claimed by several researchers [47,48]. The effect from that is the possibility that other electron production could be reduced, and thus the distortion of the electric field in transformer oil by electronic charge could also be reduced, thus increasing the breakdown voltage [29].

The plasma-treated samples show enhanced PD characteristics through the PD tests compared to pure mineral oil, untreated nanofluids, and CTAB-treated nanofluids. The presence of alumina nanoparticles in mineral oil resulted in higher viscosity than pure mineral oil. This indication was also pointed out by Jin et al. [11]. The number of PDs increases with an increasing weight percentage of alumina nanoparticles, as Makmud et al. [16] claimed. According to Jin et al.’s [14] findings, nanoparticles in mineral oil can absorb those additives such as moisture, acidity, and impurities due to oxidation on their surfaces. Consequently, the PD magnitude and PD’s number of the nanofluids were smaller than pure mineral oil, as also concluded by Jin et al. [14] and Kurimsky et al. [49]. A higher weight concentration of alumina nanoparticles can be considered worse for transformer applications based on partial discharge characteristics. A concentration above 0.1 wt% significantly influences PD magnitude and the number of PDs of the nanofluids. Agglomerated nanoparticles can explain this behavior above a certain weight concentration in mineral oil [15].

The phenomenon leading to a breakdown of liquid insulation is called a streamer, which normally consists of a positive and negative streamer. In transformer oil, the initiation of the streamer usually occurs when electrons generated by field emission at the needle tip are induced. Then, local expansion is created and forms a low-density form. As a result, electrons can speed up, and the ionization process appears to be multiplied by the charge and helps the discharge channel expand. It is noteworthy that the development of a low-density zone is indeterminate and causes different propagation traces. According to space charge theory, a positive space charge zone is generated and developed because of the enormous difference in the mobility of ions and electrons. These positive space charges distort the previous electric field distribution in the oil that the electric field at the needle tip is diminished. While the electric field at the head of the ionized zone is enhanced, this causes the ionization to occur further, and the streamer channel under positive excitation is more likely to elongate towards the ground than the negative cycles [50].

Meanwhile, the PD pattern in oil found that the negative-polarity PDs occur much more than that positive-polarity PDs. As Liu et al. [32] explained, PD with negative polarity always appears near the needle electrode with a corona-type structure. In addition, the positive ions and free electrons have been generated due to the ionization near the tip of the needle as per the space charge theory. The positive ion is moved relatively slowly in the oil, which gathered near the tip, greatly heightened, and forced the electric field between the negative tip and positive ions, causing the PD to occur much more frequently. The free electron is caused by ionization growth and is more likely to expand along the electric field line, and consequently forms a negative charge layer with penetrated distribution [51]. After all the explanations above, it can be said that the space charge effect is mainly caused by significant differences in mobility between electrons and ions [50].

A study by Yuzhen et al. [52] proved that the moisture content, acidity, and impurities due to oxidation could encourage a negative effect on the dielectric strength of mineral oil. In contrast with the PD magnitude, the pulse repetition rate is more sensitive to those additives. The additional nanoparticles added into the mineral oil can absorb those additives to their surface, henceforth contributing to the smaller number of PDs in alumina nanofluids than in the mineral oil. Based on the overall results, the plasma-treated nanoparticles in the nanofluids could have greater characterization in adsorbing those additives than the untreated samples, thus improving the PD characteristics by reducing the total discharge magnitude compared to the pure mineral oil.

The viscosities of alumina nanofluids show a similar trend at temperatures of both 40 °C and 60 °C. Adding nanoparticles into the mineral oil has created an extraordinary impact on the thermal performance of the mineral oil [53,54,55]. The highest nanoparticle concentration was 0.3wt% in alumina nanofluids, with the highest viscosity values. This behavior can be interpreted by the increased nanofluids concentration, which directly influences internal viscous shear stresses [56]. However, the increasing nanoparticle concentration could affect the viscosity and deteriorate the nanofluids’ heat transfer system [57]. A higher concentration can also contribute to the higher agglomerated cluster of nanoparticles in nanofluids, which can be considered one reason for higher viscosity [58].

Comprehensive studies in using surfactant as a stabilizer at high temperatures are still lacking. The studies of surfactants used at high temperatures are extremely important, affecting the physical properties of the surfactants and the nanoparticles [59]. Meanwhile, the excessive surfactant used in nanofluids could change the nanofluid characteristics, such as viscosity and thermal conductivity, and will be an apprehensive issue in nanofluid applications [60]. As mentioned above, this strongly supports the idea that the surfactant should be eliminated in nanofluids. Since the nanoparticle’s sizes are very small, the attractive forces between the particles can cause them to agglomerate. Furthermore, the plasma-treated nanofluids show lower viscosity values since the plasma treatment offers increased surface energy of the treated nanoparticles in mineral oil, thereby resulting in a good result with good suspension stability and lower viscosity.

Moreover, dispersing plasma-treated nano-alumina into the mineral oil has reduced the viscosity of the nanofluids compared to the untreated nanoparticles. Incorporating untreated nanoparticles with mineral oil tends to form an agglomerated dispersion of nanoparticles, which typically affects the viscosity of the nanofluids. This issue can be solved by treating the surface of nanoparticles using plasma discharge. The interfacial region formed through the plasma functionalization technique would improve the compatibility between nanoparticles and mineral oil molecules [20]. The enhancement of surface compatibility may result in enhancing the distribution of nanoparticles into mineral oil uniformly. The uniform dispersion of nanoparticles into the mineral oil would cause the viscosity of the nanofluids to reduce. In addition, the viscosity of nanofluids is also affected by the number of well-dispersed nanoparticles.

Meanwhile, the contents of additive materials such as nanoparticles have also influenced the viscosity of the nanofluids. However, plasma treatment is an alternative method proven in this study to reduce the viscosity of the nanofluids incorporated with a particular quantity of nanoparticles. The results also showed that the behavior of nanofluids below 0.3 wt% represents Newtonian fluids. In contrast, the viscosity behavior of nanofluids with 0.3 wt% nanoparticles caused them to start to become non-Newtonian fluids. Plasma-treated alumina has an effective ability in improving the viscosity of nanofluids compared to other nanoparticles because it has a very weak shear rate dependence of their viscosity. Besides, alumina is more preferable as nanoparticles due to its mass-to-volume ratio, which is among the lowest compared to other nanoparticles such as silica and titania. Thus, the plasma-treated and untreated alumina are preferred to be dispersed into the based fluids.

Based on the viscosity tests, the viscosities of both untreated and plasma-treated nanofluids have also decreased at an elevated temperature, similar to pure mineral oil. As mentioned by Jiang et al. [61], it can be elucidated that the higher the temperature, the lower the viscosity due to the increase in thermal conductivity. The downward trend in viscosity when the temperature increases can be explained by a weakening intermolecular attraction between the mineral oil and the nanoparticles [57]. Additionally, higher temperature also influenced the Brownian motion of nanoparticles and decreased nanofluid viscosity [58]. Thus, it has been found that viscosity depends strongly on both temperature and concentration. Furthermore, a previous research work stated that the formation of nanofluids has no significant effect upon the viscous resistance, but viscosity might have affected the development of streamers in that insulating liquid [62].

Furthermore, the thermal conductivity of mineral oil was enhanced with the addition of plasma-treated nanoparticles. The thermal conductivity of alumina nanofluids increased with the increase in nanoparticle concentrations and temperatures. In line with the results obtained by Bao et al. [63], higher nanoparticle surfaces, nanoparticle interaction, nanoparticle cluster, and Brownian motion of nanoparticles are among the significant factors that contributed to the thermal conductivity enhancement in alumina nanofluids when the nanoparticle concentrations were increased accordingly. In addition, the thermal conductivity of alumina nanofluids also increased with temperature, and this justification agreed with the results reported by Shah et al. [64]. This condition could be explained by the fact that the increased temperature results in decreased viscosity, contributing to the Brownian motion to escalate and thus affect the convection process in nanofluids [58]. The 0.1 wt% alumina with a CTAB nanofluid has higher thermal conductivity than pure mineral oil and untreated nanofluids but shows lower thermal conductivity than the plasma-treated nanofluid. Applying surfactant in nanofluids can cause the surface of the nanoparticles to be coated. It is noteworthy that the amount of surfactant in nanofluid is not limited by thermal conductivity, but the surfactant may cause physical or chemical instability problems [65].

Meanwhile, plasma-treated alumina nanofluids considerably enhance the thermal conductivity compared to other samples, including pure mineral oil, since the plasma treatment can convert the alumina nanoparticles to disperse much easier in the base oil [66]. Hence, plasma-treated alumina nanofluids exhibited better thermal properties than untreated alumina nanofluids and pure mineral oil. Plasma discharge occurred due to the ionization of the discharge gas, producing reactive species such as photons and electron clouds. The reactive species collide with the air molecules contained in the treatment chamber to form radical species, typically hydroxyl and oxygen-containing functional groups. The functionalized reactive species on the surface of nanoparticles could then react with the aluminum atoms, which produce a new and strong covalent on the interfacial region when the nanoparticles dispersed into the based fluids. This would create a new relaxation process that might reduce the transportation charge due to the formation of the interfacial region [67]. This interfacial region is key in improving insulation properties due to its role in trapping the charges distributed in the nanofluids. The mechanism of trapping the charges would reduce the accumulated space charge and eventually minimize the distortion of the local electric field [68]. This brings positive implications to the insulation properties, such as improving the breakdown strength and the partial discharge resistance of the nanofluids.

## 5. Conclusions

The electrical properties, dispersion stability, and thermal properties of mineral oil-based alumina nanofluids have been improved using cold atmospheric pressure plasma treatment. The easily operated atmospheric pressure plasma method has been successfully implemented to modify the surface of alumina nanoparticles. Their morphological analysis showed an improvement in the structure of the nanoparticle, according to the TEM analysis. In addition, the effect of ion and electron bombardment onto the surface of the nanoparticles can be regarded as changes in morphology. Therefore, the plasma treatment of the nanoparticles has resulted in a homogeneous dispersion and stability in nanofluids as this treatment provided stronger interactions between the mineral oil and the nanoparticles.

The electrical performances of mineral oil-based nanofluids with alumina nanoparticles have been successfully investigated and analyzed. The addition of alumina could help in improving the electrical properties of conventional mineral oil. The CTAB was chosen as a surfactant used for comparison purpose with the untreated and plasma-treated nanofluids. On top of that, the surfactant is one of the traditional methods to modify nanoparticles’ surface in order to reduce the agglomerated clusters in nanofluids. However, it has been found that it has retained many drawbacks. The 0.1 wt% alumina nanofluids recorded the highest breakdown strength as compared to 0 wt%, 0.01 wt%, 0.05 wt%, and 0.3 wt% nanofluids, respectively. It can be concluded that the increase in the weight percentage of nanoparticles up to about 0.1 wt% could increase the breakdown voltage of the nanofluids, but samples show the reduced breakdown strength at a higher weight percentage (0.3 wt%) due to the agglomerated nanoparticles. The plasma-treated samples of alumina nanofluids have been proved to have better breakdown strength than pure mineral oil, untreated samples, and CTAB-treated nanofluids. The results were also supported by the Weibull analysis, with the same trend of improvement.

Meanwhile, partial discharge characteristics of alumina-based nanofluids were performed at the applied voltage of 20 kVrms. PD magnitude and the number of PDs were depicted, and the results showed that the plasma-treated samples had lower PD magnitude and a lesser total number of PDs compared to the untreated and CTAB-treated nanofluids. Hence, plasma-treated nanofluids are believed to have excellent potential to be used as power transformer oil in practice. In short, electrical tests showed an improved breakdown strength and PD characteristics of the plasma-treated alumina-based nanofluids. The mineral oil-based nanofluids with 0.1 wt% of plasma-treated alumina showed great improvements in electrical performances. A higher concentration of more than 0.1 wt% has resulted in deteriorated breakdown strength and PD resistance characteristics.

A higher percentage of nanoparticles used in a sample has resulted in a higher viscosity as well. However, the plasma-treated samples showed lower viscosity than untreated nanofluids and CTAB-treated nanofluids with an increase in temperature, thus showing that the plasma-treated nanofluids also had excellent heat transfer properties. Additionally, the higher the alumina nanoparticles added to the base fluid, the higher the thermal conductivity of the nanofluids. Based on the results obtained, the thermal conductivity was enhanced with the increase in temperature. This behavior can be explained by the Brownian motion of the nanoparticles in the nanofluids. Besides, 0.1 wt% of plasma-treated alumina nanofluids have also shown better improvements in thermal and dispersion stability. Furthermore, CTAB as a surfactant did not help much in solving the agglomeration issue. Therefore, with the elimination of the surfactant, plasma treatment can be an alternative and environmentally friendly method to modify the surface of the nanoparticles without using any chemical solutions.

Furthermore, the important conclusion for future research is to identify the effects of plasma treatment on the zeta potential and the stability of the nanoparticles dispersed in the base fluids. In addition, the effect of plasma treatment on the pH value of nanofluids could not be neglected. Therefore, it ought to be covered in future works in order to characterize the significance of plasma treatment in enhancing the insulation and thermophysical properties of plasma treatment nanofluids.

## Figures and Tables

**Table 1:** Total enhancement of Al_2_O_3_ nanofluid samples over pure mineral oil.

Samples	Average BDV (kV)	Std. Dev	Total Enhancement (%)
0.01 wt% untreated nanofluid (UNF)	43.05	3.05	25.876
0.01 wt% plasma-treated nanofluid (PTNF)	47.47	1.54	32.778
0.05 wt% UNF	46.35	3.17	31.154
0.05 wt% PTNF	54.35	2.42	41.288
0.1 wt%UNF	55.77	1.51	42.783
0.1 wt% PTNF	58.28	0.92	45.247
0.1 wt% cetyl trimethylammonium bromide nanofluids (CTABNF)	55.80	0.99	42.813
0.3 wt% UNF	48.37	2.89	34.029
0.3 wt% PTNF	53.30	1.52	40.131

**Table 2:** Shape and scale values of Weibull analysis for alumina nanofluids.

Alumina Nanofluid Samples	Shape	Scale (kV)
0.01 wt% UNF	16.55	44.53
0.01 wt% PTNF	25.81	48.33
0.05 wt% UNF	13.78	48.02
0.05 wt% PTNF	24.73	55.57
0.1 wt%UNF	37.15	56.57
0.1 wt% PTNF	71.10	58.75
0.1 wt% + CTAB NF	60.72	56.33
0.3 wt% UNF	17.39	49.48
0.3 wt% PTNF	32.41	54.13

**Table 3:** Comparison of the present experimental data of the types of nanofluids and effective loading of nanoparticles in enhancing AC breakdown voltage.

Author/Year	Nanofluid	Effective Loading of Nanoparticle, (wt%)
Sartoratto et al./2005 [31]	Fe_2_O_3_/mineral oil	0.016
Du et al./2011 [29]	TiO_2_/mineral oil	0.075
Liu et al./2012 [32]	SiO_2_/mineral oil	0.0074
Hanai et al./2013 [33]	ZnO/mineral oil	0.0005
Rafiq et al./2016 [34]	Fe_3_O_4_/mineral oil	0.04
Baharuddin et al./2019 [35]	Al_2_O_3_/mineral oil	0.1
Present Data	Al_2_O_3_/mineral oil	0.1
Present Data	Al_2_O_3_/mineral oil	0.1

**Table 4:** Comparison of the present experimental data of type nanofluids and effective loading of nanoparticles in respect to PD magnitude.

Author/Year	Nanofluid	Effective Loading of Nanoparticle, (wt%)
Muangpratoom et al./2018 [37]	BaTiO_3_/mineral oil	0.03
Makmud et al./2019 [16]	Fe_3_O_4_/ester oil	0.01
Atiya et al./2020 [40]	Al_2_O_3_/mineral oil	0.01
Jacob et al./2019 [39]	Al_2_O_3_/mineral oil	0.03
Present Data	Al_2_O_3_/mineral oil	0.01

## Data Availability

Not applicable.

## References

[1] Yuliastuti E. (2010). Analysis of Dielectric Properties Comparison Between Mineral Oil and Synthetic Ester Oil. Master’s Thesis.

[2] Ahmad M.H., Ahmad H., Bashir N., Arief Y.Z., Malek Z.A., Kurnianto R., Yusof F. (2012). A New Statistical Approach for Analysis of Tree Inception Voltage of Silicone Rubber and Epoxy Resin under AC Ramp Voltage. Int. J. Electr. Eng. Inform..

[3] Hosier I.L., Guushaa A., Vaughan S., Swingler S.G. (2009). Selection of a suitable vegetable oil for high voltage insulation applications. J. Phys. Conf. Ser..

[4] Ahmadi M.H., Mohseni-Gharyehsafa B., Ghazvini M., Goodarzi M., Jilte R., Kumar R. (2019). Comparing various machine learning approaches in modeling the dynamic viscosity of CuO/water nanofluid. J. Therm. Anal. Calorim..

[5] Bagherzadeh S.A., D’Orazio A., Karimipour A., Goodarzi M., Bach Q.V. (2019). A novel sensitivity analysis model of EANN for F-MWCNTs–Fe_3_O_4_/EG nanofluid thermal conductivity: Outputs predicted analytically instead of numerically to more accuracy and less costs. Phys. A Stat. Mech. Appl..

[6] Peng Y., Parsian A., Khodadadi H., Akbari M., Ghani K., Goodarzi M., Bach Q.-V. (2020). Develop optimal network topology of artificial neural network (AONN) to predict the hybrid nanofluids thermal conductivity according to the empirical data of Al_2_O_3_-Cu nanoparticles dispersed in ethylene glycol. Phys. A Stat. Mech. Appl..

[7] Ghasemi A., Hassani M., Goodarzi M., Afrand M., Manafi S. (2019). Appraising influence of COOH-MWCNTs on thermal conductivity of antifreeze using curve fitting and neural network. Phys. A Stat. Mech. Appl..

[8] Giwa S., Sharifpur M., Goodarzi M., Alsulami H., Meyer J.P. (2021). Influence of base fluid, temperature, and concentration on the thermophysical properties of hybrid nanofluids of alumina-ferrofluid: Experimental data, modeling through enhanced ANN, ANFIS, and curve fitting. J. Therm. Anal. Calorim..

[9] Alrashed A.A., Gharibdousti M.S., Goodarzi M., de Oliveira L.R., Safaei M.R., Filho E.P.B. (2018). Effects on thermophysical properties of carbon based nanofluids: Experimental data, modelling using regression, ANFIS and ANN. Int. J. Heat Mass Transf..

[10] Mansour D.E.A., Elsaeed A.M. Heat transfer properties of transformer oil-based nanofluids filled with Al_2_O_3_ nanoparticles. Proceedings of the IEEE International Conference on Power and Energy (PECon).

[11] Jin H., Andritsch T., Morshuis P.H.F., Smit J.J. AC breakdown voltage and viscosity of mineral oil based SiO_2_ nanofluids. Proceedings of the Conference on Electrical Insulation and Dielectric Phenomena—Annual Report (CEIDP).

[12] Sridhara V., Satapathy L.N. (2011). Al_2_O_3_-based nanofluids: A review. Nanoscale Res. Lett..

[13] Du Y.F., Lv Y.Z., Zhou J.Q., Li X.X., Li C.R. Breakdown properties of transformer oil—based TiO_2_ nanofluid. Proceedings of the Conference on Electrical Insulation and Dielectric Phenomena—Annual Report (CEIDP).

[14] Jin H., Morshuis P., Mor A.R., Smit J.J., Andritsch T. (2015). Partial discharge behavior of mineral oil based nanofluids. IEEE Trans. Dielectr. Electr. Insul..

[15] Prasad D., Chandrasekar S. (2017). Effect of Nano-SiO_2_ Particles on Partial Discharge Signal Characteristics of FR_3_ Transformer Oil. J. Adv. Chem..

[16] Makmud M.Z.H., Illias H.A., Chee C.Y., Dabbak S.Z.A. (2019). Partial Discharge in Nanofluid Insulation Material with Conductive and Semiconductive Nanoparticles. Materials.

[17] Mehrali M., Sadeghinezhad E., Latibari S.T., Kazi S.N., Mehrali M., Zubir M.N.B.M. (2014). Investigation of Thermal Conductivity and Rheological Properties of Nanofluids Containing Graphene Nanoplatelets. Nanoscale Res. Lett..

[18] Dong M., Shen L.P., Wang H.B., Miao J. (2013). Investigation on the Electrical Conductivity of Transformer Oil-Based AlN Nanofluid. J. Nanomater..

[19] Musa F.N., Bashir N., Ahmad M.H., Buntat Z., Piah M.A.M. (2016). Investigating the Influence of Plasma-Treated SiO_2_ Nanofillers on the Electrical Treeing Performance of Silicone-Rubber. Appl. Sci..

[20] Yan W. (2013). Nanocomposite Dielectric Materials for Power System Equipment. Ph.D. Thesis.

[21] Awang N.A., Ahmad M.H., Arief Y.Z., Zakaria I.H., Ahmad N. (2017). The Effect of Plasma-Treated Boron Nitride on Partial Discharge Characteristics of LDPE. Int. J. Electr. Comput. Eng..

[22] Yan W., Han Z.J., Phung B.T., Ostrikov K. (2012). Silica Nanoparticles Treated by Cold Atmospheric-Pressure Plasmas Improve the Dielectric Performance of Organic-Inorganic Nanocomposites. ACS Appl. Mater. Interfaces.

[23] Kim Y.J., Yu Q., Ma H. (2014). Plasma Treatment of Nanoparticles and Carbon Nanotubes for Nanofluids. Encycl. Microfluids Nanofluids.

[24] Singh M., Kundan L. (2013). Experimental Study on Thermal Conductivity and Viscosity of Al_2_O_3_-Nanotransformer Oil. Int. J. Theor. Appl. Res. Mech. Eng. Nanofluids.

[25] Kong P. Atmospheric-Pressure Plasma Process and Applications. Proceedings of the Sohn International Symposium on Advanced Processing of Metals and Materials, Principles, Technologies, and Industrial Practice.

[26] Tendero C., Tixier C., Tristant P., Desmaison J., Leprince P. (2006). Atmospheric pressure plasmas: A review. Spectrochim. Acta Part B Spectrosc..

[27] Lv Y., Wang W., Ma K., Zhang S., Zhou Y., Li C., Wang Q. Nanoparticle Effect on Dielectric Breakdown Strength of Transformer Oil-Based Nanofluids. In Proceeding of the Annual Report Conference on Electrical Insulation and Dielectric Phenomena (CEIDP).

[28] Zhou Y., Sui S.Y., Li J., Wang Z.Y., Cui W., Lv Y.Z., Li C.R. (2018). Statistical analysis of moisture’s effect on AC breakdown strength of TiO_2_ nanofluids. J. Mol. Liq..

[29] Du Y., Lv Y., Li C., Chen M., Zhou J., Li X., Zhou Y., Tu Y. (2011). Effect of electron shallow trap on breakdown performance of transformer oil-based nanofluids. J. Appl. Phys..

[30] Abernethy R.B. (2006). The New Weibull Handbook—Reliability and Statistical Analysis for Predicting Life, Safety, Supportability, Risk, Cost and Warranty Claims.

[31] Sartoratto P.P.C., Neto A.V.S., Lima E.C.D., De Sá A.L.C.R., Morais P.C. (2005). Preparation and electrical properties of oil-based magnetic fluids. J. Appl. Phys..

[32] Liu J., Zhou L., Wu G., Zhao Y., Liu P., Peng Q. (2012). Dielectric frequency response of oil-paper composite insulation modified by nanoparticles. IEEE Trans. Dielectr. Electr. Insul..

[33] Hanai M., Hosomi S., Kojima H., Hayakawa N., Okubo H. Dependence of TiO_2_ and ZnO nanoparticle concentration on electrical insulation characteristics of insulating oil. Proceedings of the Conference on Electrical Insulation and Dielectric Phenomena—Annual Report (CEIDP).

[34] Rafiq M., Li C., Ge Y., Lv Y., Yi K. Effect of Fe_3_O_4_ nanoparticle concentrations on dielectric property of transformer oil. Proceedings of the IEEE International Conference on High Voltage Engineering and Application (ICHVE).

[35] Baharuddin M.F., Zakaria I.H., Ahmad M.H., Aulia, Nawawi Z., Sidik M.A.B., Jambak M.I. (2019). Effect of Surfactant on Breakdown Strength Performance of Transformer Oil-Based Nanofluids. J. Electr. Eng. Technol..

[36] Nagendran S., Chandrasekar S. (2018). Investigations on partial discharge, dielectric and thermal characteristics of nano SiO_2_ modified sunflower oil for power transformer applications. J. Electr. Eng. Technol..

[37] Muangpratoom P., Pattanadech N. (2018). Breakdown and Partial discharge characteristics of Mineral oil-based nanofluids. IET Sci. Meas. Technol..

[38] Mohamad N., Azis N., Jasni J., Kadir M., Yunus R., Yaakub Z. (2021). Experimental Study on the Partial Discharge Characteristics of Palm Oil and Coconut Oil Based Al_2_O_3_ Nanofluids in the Presence of Sodium Dodecyl Sulfate. Nanomaterials.

[39] Jacob J.S., Dhanya L., Tharamal A., Avinash N., Preetha P. Partial Discharge Characteristics of Nanofilled Mineral Oil. Proceedings of the IEEE Region 10 Symposium (TENSYMP).

[40] Atiya E.G., Mansour D.E.A., Izzularab M.A. (2020). Partial discharge development in oil-based nanofluids: Inception, propagation and time transition. IEEE Access.

[41] ISO—ISO 3104:2020—Petroleum Products—Transparent and Opaque Liquids—Determination of Kinematic Viscosity and Calculation of Dynamic Viscosity. https://www.iso.org/standard/67965.html.

[42] Yu Q., Kim Y.J., Ma H. (2008). Nanofluids with plasma treated diamond nanoparticles. Appl. Phys. Lett..

[43] Wong K.-F.V., Kurma T. (2008). Transport properties of alumina nanofluids. Nanotechnology.

[44] Xiang D., Shen L., Wang H. (2019). Investigation on the Thermal Conductivity of Mineral Oil-Based Alumina/Aluminum Nitride Nanofluids. Materials.

[45] Hwang Y., Lee J.-K., Lee J.-K., Jeong Y.-M., Cheong S.-I., Ahn Y.-C., Kim S.H. (2008). Production and dispersion stability of nanoparticles in nanofluids. Powder Technol..

[46] Martin R. (2010). Experiences in Service with New Insulating Liquids.

[47] Wang Z., Zhou Y., Lu W., Peng N., Chen W. (2019). The Impact of TiO_2_ Nanoparticle Concentration Levels on Impulse Breakdown Performance of Mineral Oil-Based Nanofluids. Nanomaterials.

[48] Rafiq M., Li C., Khan I., Zhifeng H., Lv Y., Yi K. Preparation and breakdown properties of mineral oil-based alumina nanofluids. Proceedings of the International Conference on Emerging Technologies (ICET).

[49] Kurimsky J., Rajnak M., Cimbala R., Paulovicova K., Rozynek Z., Kopcansky P., Timko M. (2021). Electrical discharges in ferrofluids based on mineral oil and novel gas-to-liquid oil. J. Mol. Liq..

[50] Li Y., Wen J.Y., Liang Y., Wu J., Qin S., Zhang G.J. Streamer Discharge Propagation and Branching Characteristics in Transformer Oil under AC Voltage: Partial Discharge and Light Emission. Proceedings of the IEEE 19th International Conference on Dielectric Liquids (ICDL).

[51] Massimo P., Carlo M., Ray B. (2006). PD Pulse Burst Characteristics of Transformer Oils. IEEE Trans. Power Deliv..

[52] Lv Y.Z., Zhou Y., Li C.R., Wang Q., Qi B. (2014). Recent progress in nanofluids based on transformer oil: Preparation and electrical insulation properties. IEEE Electr. Insul. Mag..

[53] Jin H., Andritsch T., Tsekmes I.A., Kochetov R., Morshuis P., Smit J.J. (2014). Properties of Mineral Oil based Silica Nanofluids. IEEE Trans. Dielectr. Electr. Insul..

[54] Zhang Y., Ho S., Fu W. (2018). Heat Transfer Comparison of Nanofluid Filled Transformer and Traditional Oil-Immersed Transformer. AIP Adv..

[55] Bobbo S., Colla L., Scattolini M., Agresti F., Barison S., Pagura C., Fedele L. (2011). Thermal Conductivity and Viscosity Measurements of Water-Based Silica Nanofluids. NSTI Nanotech..

[56] Nguyen C., Desgranges F., Galanis N., Roy G., Maré T., Boucher S., Mintsa H.A. (2008). Viscosity data for Al_2_O_3_—Water nanofluid—Hysteresis: Is heat transfer enhancement using nanofluids reliable?. Int. J. Therm. Sci..

[57] Mishra P.C., Mukherjee S., Nayak S.K., Panda A. (2014). A brief review on viscosity of nanofluids. Int. Nano Lett..

[58] Mostafizur R.M., Saidur R., Abdul A.A.R., Bhuiyan M.H.U. (2015). Thermophysical properties of methanol—Based Al_2_O_3_ nanofluids. Int. J. Heat Mass Transf..

[59] Koca H.D., Doganay S., Turgut A., Tavman I.H., Saidur R., Mahbubul I.M. (2018). Effect of particle size on the viscosity of nanofluids: A review. Renew. Sustain. Energy Rev..

[60] Kim Y.J., Yu Q., Ma H. (2008). Plasma for Nanofluids. Encyclopedia of Microfluidics and Nanofluidics.

[61] Jiang H., Hou X., Su D., Liu H., Ali M.K.A. (2021). Elucidation of the thermophysical mechanism of hexagonal boron nitride as nanofluids additives. Adv. Powder Technol..

[62] Li D., Xie W., Fang W. (2011). Preparation and properties of copper-oil-based nanofluids. Nanoscale Res. Lett..

[63] Bao L., Zhong C., Jie P., Hou Y. (2019). The effect of nanoparticle size and nanoparticle aggregation on the flow characteristics of nanofluids by molecular dynamics simulation. Adv. Mech. Eng..

[64] Shah J., Ranjan M., Davariya V., Gupta S.K., Sonvane Y. (2017). Temperature-dependent thermal conductivity and viscosity of synthesized α-alumina nanofluids. Appl. Nanosci..

[65] Choi C., Yoo H., Oh J. (2008). Preparation and heat transfer properties of nanoparticle-in-transformer oil dispersions as advanced energy-efficient coolants. Curr. Appl. Phys..

[66] Wiken R. (2010). Plasma Treatment of Microparticles and Nanoparticles at Atmospheric Pressure Permits New Materials and Applications. Ann. Rep. Adv. Mater..

[67] Montanari G.C., Palmieri F., Testa L., Motori A., Saccani A., Patuelli F. (2006). Polarization processes of nanocomposite silicate-EVA and PP materials. IEEJ Trans. Fundam. Mater..

[68] Saman N.M., Ahmad M.H., Buntat Z. (2021). Application of Cold Plasma in Nanofillers Surface Modification for Enhancement of Insulation Characteristics of Polymer Nanocomposites: A Review. IEEE Access.

